# Distributed functions of detection and discrimination of vibrotactile stimuli in the hierarchical human somatosensory system

**DOI:** 10.3389/fnhum.2014.01070

**Published:** 2015-01-21

**Authors:** Junsuk Kim, Klaus-Robert Müller, Yoon Gi Chung, Soon-Cheol Chung, Jang-Yeon Park, Heinrich H. Bülthoff, Sung-Phil Kim

**Affiliations:** ^1^Department of Brain and Cognitive Engineering, Korea UniversitySeoul, South Korea; ^2^Machine Learning Group, Berlin Institute of TechnologyBerlin, Germany; ^3^Department of Global Biomedical Engineering, IBS Center for Neuroscience Imaging Research, Sungkyunkwan UniversitySuwon, South Korea; ^4^School of Biomedical Engineering, Konkuk UniversityChungju, South Korea; ^5^Department of Human Perception, Cognition and Action, Max Planck Institute for Biological CyberneticsTübingen, Germany; ^6^Department of Human and Systems Engineering, Ulsan National Institute of Science and TechnologyUlsan, South Korea

**Keywords:** fMRI, vibrotactile stimulation, somatosensory cortex, functional specialization, hierarchical tactile processing

## Abstract

According to the hierarchical view of human somatosensory network, somatic sensory information is relayed from the thalamus to primary somatosensory cortex (S1), and then distributed to adjacent cortical regions to perform further perceptual and cognitive functions. Although a number of neuroimaging studies have examined neuronal activity correlated with tactile stimuli, comparatively less attention has been devoted toward understanding how vibrotactile stimulus information is processed in the hierarchical somatosensory cortical network. To explore the hierarchical perspective of tactile information processing, we studied two cases: (a) discrimination between the locations of finger stimulation; and (b) detection of stimulation against no stimulation on individual fingers, using both standard general linear model (GLM) and searchlight multi-voxel pattern analysis (MVPA) techniques. These two cases were studied on the same data set resulting from a passive vibrotactile stimulation experiment. Our results showed that vibrotactile stimulus locations on fingers could be discriminated from measurements of human functional magnetic resonance imaging (fMRI). In particular, it was in case (a) we observed activity in contralateral posterior parietal cortex (PPC) and supramarginal gyrus (SMG) but *not* in S1, while in case; (b) we found significant cortical activations in S1 but *not* in PPC and SMG. These discrepant observations suggest the functional specialization with regard to vibrotactile stimulus locations, especially, the hierarchical information processing in the human somatosensory cortical areas. Our findings moreover support the general understanding that S1 is the main sensory receptive area for the sense of touch, and adjacent cortical regions (i.e., PPC and SMG) are in charge of a higher level of processing and may thus contribute most for the successful classification between stimulated finger locations.

## Introduction

The somatosensory system conveys mechano-sensory information via sensory afferents through the spinal cord, brainstem, and thalamus to the somatosensory cortex (Kaas, 1993; McGlone and Reilly, 2010; Kalberlah et al., 2013). In this hierarchical view of the organization of the somatosensory system (Iwamura, 1998; Bodegård et al., 2001), primary somatosensory cortex (S1) is a main sensory receptive area for the sense of touch and distributes somatic information to adjacent posterior parietal cortex (PPC) and supramarginal gyrus (SMG) for integrating different somatic sensory modalities and higher level processing. Previous lesion studies revealed that lesions of S1 impaired the detection of tactile stimuli, whereas lesions of posterior parietal lobe led to impairments of more complex functions such as shape and roughness recognition (Freund, 2003). The lesions of parietal lobe entailed tactile neglect and apraxia (Binkofski et al., 2001), in addition, Vallar et al. showed that parietal cortex has been implicated in even more complex tactile processing including tactile attention (Vallar et al., 2003). In line with the lesion studies, neuroimaging studies demonstrated that S1 is associated with the processing of tactile form (Van Boven et al., 2005; Wacker et al., 2011) and the higher level of tactile processing induced significant activations in parietal cortex as well as in somatosensory cortex (Bodegård et al., 2001; Zhang et al., 2005; Hartmann et al., 2008). Although exact mechanisms of hierarchical tactile information processing are controversial in the dynamic causal modeling (DCM) studies based on functional magnetic resonance imaging (fMRI) data (Liang et al., 2011; Kalberlah et al., 2013), recent studies (Chung et al., 2013; Kalberlah et al., 2013) provided substantial evidence for hierarchical tactile information processing in human somatosensory cortex. These previous observations guide us to form a hypothesis that somatosensory cortical processes are organized hierarchically; S1 contributes to low-level processing while adjacent cortical regions (e.g., PPC and SMG) more likely contribute to high-level processing. To verify this hypothesis, the present study focuses on somatosensory processing of information about passive vibrotactile stimulus locations. It leads us to form a more specific hypothesis on hierarchical somatosensory cortical processes that S1 contributes to the detection of a vibrotactile stimulus in a particular location while PPC and SMG contribute to the discrimination of vibrotactile stimulus locations.

We test our hypothesis by applying vibrotactile stimulations to all three phalanges of the fingers of humans while acquiring somatosensory cortical activities using 3T fMRI. When a vibrotactile stimulus was provided on one of the phalanges, for instance, one can perceive the stimulus itself, but also can identify the location of the stimulus. However, it is unknown whether these processes involve the same local brain region or not. To address this, we applied two different analytic approaches on the same data set to seek brain regions implicated in (a) discrimination between the locations of finger stimulation; and (b) detection of stimulation against no stimulation on individual fingers. The present study also focuses on the neural activities elicited by the “passive” stimulation. Despite previous findings delineating neuronal activities correlated with active touch (e.g., object exploration) (Binkofski et al., 1999; Stoeckel et al., 2003), it seems that few studies have attempted to decode information about passive tactile stimuli from neuronal activities. The analysis of fMRI data is carried out not only using the traditional univariate general linear model (GLM) analysis that finds correlations of the blood oxygen level-dependent (BOLD) signal with stimuli, but also using a more recent multivariate decoding analysis that decodes stimulation information from the BOLD signal. The univariate GLM analysis has been used to find cortical representations of somatosensory stimuli (Kurth et al., 1998, 2000). Perhaps the best example can be found in human finger somatotopy in S1 that forms a distict spatial order of finger mapping; the little finger mapping is located more medially while the thumb mapping is located more laterally in S1 (Nelson and Chen, 2008; Martuzzi et al., 2014). Multivariate decoding analyses such as multi-voxel pattern analysis (MVPA) have also been used to discriminate different sensorimotor information from measurements of fMRI using advanced machine learning techniques. For example, an fMRI study showed that the information of ipsilateral isometric finger presses in motor cortex could be decoded using an information based statistical approach (Diedrichsen et al., 2013). Nambu et al. distinguished between two different sequences of finger movements from the fMRI BOLD signal (Nambu et al., 2010). Furthermore, a multivariate decoding study reported that the touched body sites could be decoded with significantly higher accuracies than a chance level from distributed patterns of fMRI data in primary and secondary somatosensory cortical areas (Beauchamp et al., 2009).

The motivation behind using both univariate and multivariate analyses is to investigate which analysis deals better with hierarchical cortical processing with different levels of complexity. The traditional univariate analysis using GLM might be able to find brain activations related to detection of a stimulus but not be sensitive enough for more complex processes such as discrimination of stimulus locations that can involve widely distributed neuronal activities. On the other hand, the MVPA technique, used in our study for multivariate analysis, can relate distinct activity patterns within a certain brain region to stimulus parameters and accumulate the weak information available at each brain region in an efficient way (Haynes and Rees, 2006). Several studies also reported that the MVPA provided considerable increases in the amount of information compared to the univariate statistical parametric mapping (O’Toole et al., 2007; Gallivan et al., 2011). Hence, we utilize the information-based brain mapping by means of a cubical searchlight MVPA to better discriminate cortical differences between vibrotactile stimulus locations (Kriegeskorte et al., 2006).

## Materials and methods

### Participants and experimental procedures

Ten right-handed healthy volunteers (10 males, 25 ± 2.9 years old) with no history of neurological disorders participated in the study after having given written informed consent. All were right-handed and no participant reported to have deficits in tactile processing. Experimental procedures were approved by the Korea University Institutional Review Board (KU-IRB-11-46-A-1) and the study was conducted in accordance with the Declaration of Helsinki.

**Figure 1:**
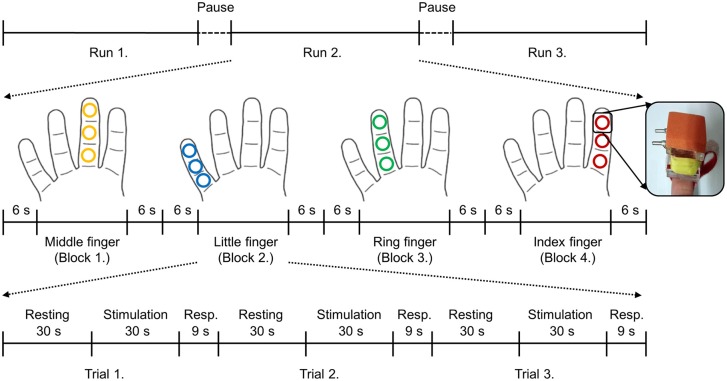
**Brief sketch of the experimental design and the stimulation device**. Each trial consisted of three periods: resting (30 s), stimulation (30 s), and response (9 s). No stimulation was applied during the resting period and 200 Hz of vibrotactile stimulation was applied on the one of three segments of each finger in a random order during the stimulation period. After the finger stimulation, participants were asked to press the button with their left hand if they perceived the stimulation regardless of the stimulus locations. Stimulated locations of each finger are depicted in the same color. Even though each finger was stimulated at three different sites, those were considered as the same finger locations in this study.

The vibrotactile stimuli were delivered to each digit (four digits: index, middle, ring, and little) of the right hand using independently controlled MR compatible devices with the size of 10 × 10 mm^2^ at the following three sites: the distal (tip), medial (middle), and proximal (base) phalanx (Figure 1). This planar-coil-type actuator, which used a planar coil instead of conventional electric wire, generated vibrating stimulation through interaction of the current of the planar coil with the static magnetic field of the MR scanner (Kim et al., 2013). The stimulation strength was set as 330 mV and the frequency was set to 200 Hz. From the pilot study, we empirically explored various strengths of the vibrotactile stimuli and chose the strength of 330 mV that could be sustained consistent in the MR field and clearly detected by the participants. These vibrotactile stimuli elicit a sense of vibration mainly transduced by Pacinian corpuscles (Johansson and Flanagan, 2009; Chung et al., 2013). During the functional image acquisition, the participants lied supine in the scanner with their eyes closed and wore a headset to prevent disturbances from the surroundings. Participants performed 3 fMRI runs and short breaks were provided for about 3 min between runs (Figure 1). Within each run, 12 trials were presented in 4 blocks (stimulation of each finger was considered as one block) of 3 trials. Each block started and ended with a 6 s of baseline period, and stimulated finger order was pseudo-randomized between participants. Each trial was made up of three consecutive periods; a resting period of 30 s followed by a stimulation period of 30 s plus a response period of 9 s. In the stimulation period, vibrotactile stimulus was applied on one of three segments of each finger of the right hand, and no stimulation was applied in the resting period. Each run lasted 12 min 56 s. Over the entire experiment, each stimulated phalanx was stimulated 3 times. Although the fingertips were assumed to be more sensitive than the middle and base of the finger (Johansson and Vallbo, 1979), each finger was equally stimulated at three different sites because the current study focused on inter-finger location discrimination rather than within-finger locations. During the fMRI scanning, we asked participants to press the button after the presentation of each stimulus using their left hand if they had perceived a vibrotactile stimulus on their finger and all participants responded that they have felt each stimulation.

### Data acquisition and preprocessing

Neuroimaging data were acquired using a 3T MRI system (Magnetom TrioTim, Siemens Medical Systems, Erlangen, Germany) equipped with a standard 32-channel head coil. Anatomical images were obtained using a T1-weighted 3D MPRAGE sequence with repetition time (TR) = 1,900 ms, echo time (TE) = 2.48 ms, flip angle = 9°, field of view (FOV) = 200 mm, and spatial resolution = 0.8 × 0.8 × 1 mm^3^. 35 axial functional images were obtained using a T2^*^-weighted gradient echo-planar imaging (EPI) sequence with TR = 3,000 ms, TE = 30 ms, flip angle = 90°, FOV = 192 mm, acquisition matrix = 128 × 128, slice thickness = 2 mm, and in-plane resolution = 1.5 × 1.5 mm^2^. The coverage of functional images was the whole depth of the somatosensory area. Standard preprocessing of the fMRI data were performed using SPM8 (Wellcome Department of Imaging Neuroscience, UCL, London, UK). The EPI data were corrected for slice-timing differences, realigned for motion correction, co-registered to the individual T1-weighted images, and spatially smoothed by a 4 mm full-width-half-maximum (FWHM) Gaussian kernel. Data was analyzed in native subject space; only the performance maps resulting from the individual searchlight analyses were normalized to the Montreal Neurological Institute (MNI) space.

### Vibrotactile stimulus location decoding

An information-based analysis with a cubical searchlight was used to find spatially localized neuronal patterns differing across vibrotactile stimulus locations (Kriegeskorte et al., 2006). Specifically, the searchlight MVPA was performed on parameter estimates (i.e., model coefficients) that were extracted from a GLM. Parameter estimates explained how much the stimulus location variable contributed to the variation of neuronal signals and have been utilized as input features to the searchlight MVPA in previous studies (Peelen et al., 2010; Hebart et al., 2012). For the GLM, a standard predictor was made by the convolution of a box-car function of the stimulation “on” periods with a standard model of the hemodynamic response function (HRF) of SPM8. We implemented a GLM independently for each stimulation condition. A total of 36 regressors (3 trials × 4 blocks × 3 runs) were acquired in each participant. Regressors were fitted to each voxels and the resulting parameter estimates were used as input features to the MVPA. Through a searchlight analysis, multi-voxel activation patterns surrounding each voxel were measured within a three-voxel radius cubical searchlight (i.e., a cubic of 7^3^ = 343 adjacent voxels including itself) and a Gaussian Naive Bayes (GNB) classifier predicted the tactile stimulus location among the four fingers. Decoding accuracy evaluated by a 5-fold cross validation procedure (in a leave-one-block-out paradigm) was allocated to the center voxel of each searchlight. The resulting decoding accuracy value stored in each voxel was corrected by subtracting chance-level accuracy (i.e., 25% in this case). After mapping the decoding accuracy onto every voxel, we generated each participant’s spatially normalized (to MNI space) brain mask of decoding accuracies. A random-effects group analysis (*N* = 10) was carried out on the single-subject accuracy maps to establish commonalities among individual neural representations. This test was implemented as a one-sample *t*-test against 0 to identify above-chance decoding accuracy in the MVPA.

To correct the searchlight cluster results for multiple comparisons, we employed the method described by Oosterhof et al. (2010). We compared the size of the clusters resulted from the group analysis to a reference distribution of clusters that one would obtain by chance. If there is no real effect, the sign of the searchlight accuracy values would be “+” or “−” with equal probability of 50% (which is allowed under the null hypothesis of chance accuracy). To identify how large clusters would be determined when the null hypothesis is true, we sampled from the searchlight results maps and randomly flipped the sign of the maps of a random number of participants. These maps were then considered one group sample from the null effect case, and a random-effect analysis on these maps calculated the size of the biggest cluster. This procedure was repeated 1000 times and the computed cluster sizes for each iteration were collected, yielding the distribution of cluster sizes under the null hypothesis. In this study, we reported the clusters in the 5% of upper tail (i.e., *p* < 0.05 corrected for multiple comparisons via cluster size).

Additionally, a univariate group GLM analysis was performed, to determine whether the voxel-wise fMRI responses contain information that would allow discrimination between stimulations on different fingers on the right hand. We probed activation patterns associated with BOLD signal differences between “one finger” and “other three fingers” stimulation conditions. In other words, stimulation blocks for each finger were contrasted against the stimulation blocks for the other three fingers. With this contrast, we assumed that the group GLM analysis would reveal the brain areas reflecting the distinct pattern of each finger from the other fingers.

### Contrasting vibrotactile stimulation against no stimulation

The searchlight MVPA as well as the univariate GLM analysis was carried out to seek brain regions, which play a role in determining whether the stimulation was applied. Unlike stimulus locations decoding analysis, this contrast analysis derived activation patterns related to the detection of vibrotactile stimulation. The searchlight MVPA was performed to discriminate two classes (i.e., finger stimulation vs. no stimulation) for each of four fingers. Then the aforementioned second-level group analysis was performed again to find the common significant voxel clusters across all the participants. For the univariate group GLM analysis, we employed a subtraction method (stimulation phase—resting phase) to yield common activated areas for each stimulated finger.

## Results

### Vibrotactile stimulus location decoding

**Figure 2:**
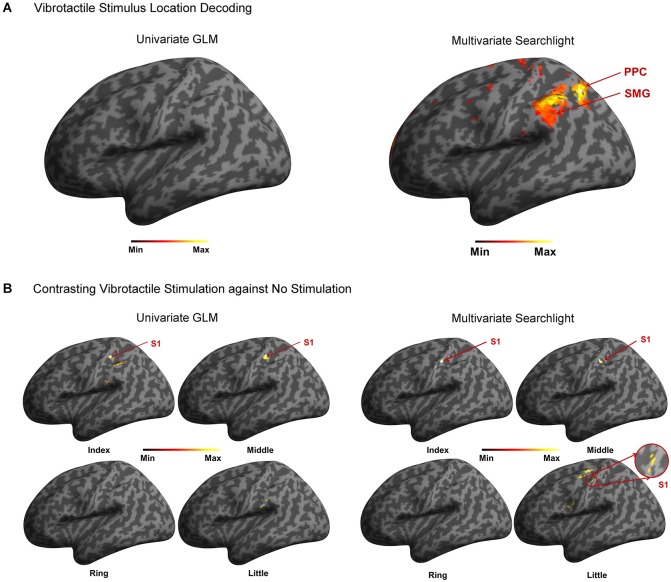
**Summary of the analysis. (A)** In the stimulated finger decoding analysis, searchlight MVPA found two significant clusters located in contralateral PPC and SMG. However, no significant cluster was found from univariate GLM approach. **(B)** In the contrast analysis of finger stimulation vs. no stimulation, significant clusters in contralateral S1 were observed except ring finger stimulation using searchlight MVPA. On the other hand, univariate GLM revealed the contralateral S1 activations in response to stimulation on the index and middle fingers.

**Figure 3:**
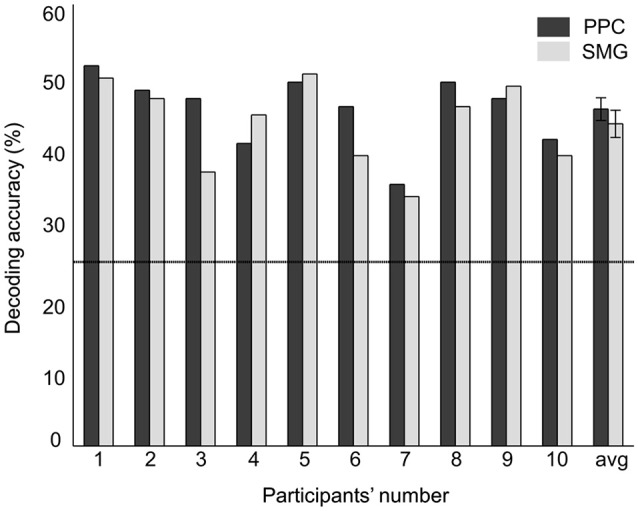
**Decoding accuracies of each significant cluster from searchlight MVPA**. The rightmost value indicates the average decoding accuracy across all the participants. Error bars indicate standard errors and a chance level is marked by the dashed line (25%).

A random-effects group analysis with the searchlight MVPA resulted in two distinct clusters exhibiting statistically significant decoding capabilities to predict a stimulated finger location from the BOLD signal (*p* < 0.05 FWE, cluster size >10) (Figure 2A). The first cluster was located in contralateral PPC and the second cluster in contralateral SMG. Note that no significant cluster was found in S1 from the searchlight MVPA. Table 1 shows the MNI coordinates, cluster sizes, peak *t*-values, and peak *z*-values for those significant clusters. The clusters we found were unlikely to have occurred by chance: a bootstrap procedure (Oosterhof et al., 2010) revealed that the probabilities of obtaining a cluster as large as ours were <0.05. Therefore, our clusters remained significant after the correction for multiple comparisons (Nichols and Hayasaka, 2003; Oosterhof et al., 2010). Decoding accuracies from the voxels of each cluster were significantly higher than the chance level in every participant (Figure 3). Accuracies from each significant cluster were as follows (given in mean ± standard deviation, highest and lowest accuracy for each cluster): 45.8 ± 4.9%, 51.7% and 35.6% for the contralateral PPC cluster; 43.8 ± 5.9%, 50.6%, and 33.9% for the contralateral SMG cluster. A one sample *t*-test verified that decoding accuracy results significantly exceeded the chance level for both clusters (PPC: *t*_9_ = 13.42, *p* < 0.01; SMG: *t*_9_ = 10.15, *p* < 0.01). The independent two-sample *t*-test was used to test whether mean decoding accuracies were significantly different from each cluster, however, no significant difference was found (*t*_18_ = 0.83, *p* = 0.42).

**Table 1:** **Significant clusters from stimulated finger location decoding using searchlight MVPA (*p* < 0.05 FWE, cluster size >10)**.

		MNI coordinates					
Regions	Side	x	y	z	Voxels	T	Z
**Posterior parietal cortex**	**L**	**−34**	**−74**	**54**	**83**	**19.39**	**5.70**
-	L	−50	−60	54		12.77	5.05
-	L	−42	−66	56		11.83	4.92
**Supramarginal gyrus**	**L**	**−54**	**−48**	**54**	**74**	**14.87**	**5.29**
-	L	−50	−56	56		9.67	4.58
-	L	−56	−50	46		9.25	4.50

*Side indicates hemisphere (R = right, L = left), cluster size indicates N voxels, T indicates peak t-values, Z indicates peak z-values. Entries without brain region name labels indicate sub-peak within the cluster named above*.

Having found specific brain regions that provided useful information for vibrotactile stimuli classification as present above, we further investigated how regional decoding accuracy of individual participants varied with their brain signal change. To assess a correlation between the decoding accuracy and the fMRI signal, we calculated the average percentage change of the BOLD signal in each significant cluster for each participant. The average percentage change was defined as the mean percentage change of the BOLD signal intensity from a resting to a stimulation period for given voxels. Then, we correlated these average percentage changes with the decoding accuracy values for each significant cluster. The significance of the correlation coefficient was evaluated with the *F*-test. The pairwise correlation analysis showed that no significant correlation was found between decoding accuracy and BOLD signal change in PPC (*r*^2^ = 0.02, *p* = 0.73) and SMG (*r*^2^ = 0.07, *p* = 0.45).

**Figure 4:**
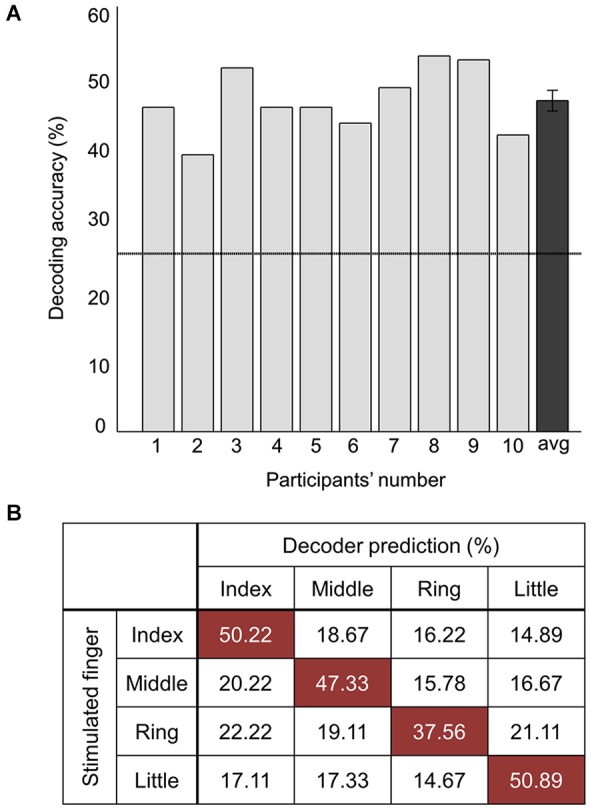
**Decoding performance combining all the voxels of significant clusters. (A)** Average decoding accuracies of ten participants from searchlight MVPA. The rightmost value indicates the average accuracy across all the participants. Error bars indicate standard errors and a chance level is marked by the dashed line (25%). **(B)** Confusion matrix for the stimulated finger decoding analysis. The rows of the matrix indicate the locations of vibrotactile stimulus provided to the participants (i.e., true label) and the columns of the matrix indicate the predictions by the decoder (i.e., predicted label). Each cell shows the percentage of correct prediction.

Decoding accuracy using all of the voxels spanning both clusters was also significantly higher than the chance level (Figure 4A). The stimulated location between fingers was decoded with a mean accuracy of 46.5 ± 3.7% across participants; the highest and lowest accuracy were 52.8% and 38.9%, respectively. One sample *t*-test revealed that accuracy for discrimination of the stimulation locations significantly surpassed the chance level (*t*_9_ = 14.77, *p* < 0.01). Figure 4B illustrates the confusion matrix resulting from the searchlight analysis using the voxels in both clusters. The decoding accuracy on a given row i and column j of the confusion matrix represents the proportion that a stimulated finger i is predicted to be a finger j (an ideal confusion matrix would have 100% everywhere on the diagonal and 0% in the off-diagonal entries). The cells of the highest accuracy in each row were observed on the diagonal entries of the confusion matrix. The decoding accuracies for the index, middle, ring, and little finger were 50.2%, 47.3%, 37.6%, and 50.9%, respectively, showing the lowest performance for decoding the location of the ring finger. Unlike the searchlight MVPA results, the GLM analysis contrasting each finger with the other three fingers did not identify any significantly activated cluster for all the fingers (Figure 2A).

### Contrasting vibrotactile stimulation against no stimulation

We investigated the cortical activation patterns with respect to the contrast of finger stimulation vs. no stimulation in both approaches; searchlight MVPA and GLM. First, the searchlight MVPA group analysis yielded significant clusters in postcentral gyrus within contralateral S1 when the stimulation was applied on the index, middle, and little fingers (*p* < 0.001 uncorrected, cluster size >10) (Figure 2B and Table 2). For the ring finger stimulation, significant clusters have been observed in the contralateral precuneus, ipsilateral supplementary motor region, and ipsilateral medial superior frontal gyrus. Next, the univariate group GLM analysis revealed activated voxel clusters in postcentral gyrus within contralateral S1 in response to stimulation on the index and middle fingers (*p* < 0.001 uncorrected, cluster size >10) (Table 3). When the stimulus was applied on the ring fingers, no cluster was activated across the whole brain. For the little finger stimulation, contralateral SMG and supplementary motor regions were activated. In summary, both multivariate and univariate results showed the similar activation patterns in contralateral S1.

**Table 2:** **Significant clusters from two-class classification (finger stimulation vs. no stimulation) using searchlight MVPA (*p* < 0.001 uncorrected, cluster size >10)**.

		MNI coordinates					
Regions	Side	x	y	z	Voxels	T	Z
*Index finger*
**Postcentral gyrus**	**L**	**−36**	**−34**	**62**	**21**	**5.27**	**3.47**
-	L	−44	−26	62		4.95	3.36
**Postcentral gyrus**	**L**	**−54**	**−20**	**48**	**19**	**5.08**	**3.40**
*Middle finger*
**Postcentral gyrus**	**L**	**−50**	**−20**	**60**	**12**	**5.30**	**3.48**
**Postcentral gyrus**	**L**	**−48**	**−26**	**50**	**21**	**5.14**	**3.43**
-	L	−42	−28	44		4.69	3.25
*Ring finger*
**Precuneus**	**L**	**0**	**−62**	**58**	**202**	**6.32**	**3.81**
**Supplementary motor area**	**R**	**8**	**4**	**76**	**61**	**6.10**	**3.75**
**Medial superior frontal gyrus**	**R**	**6**	**68**	**22**	**198**	**5.79**	**3.65**
*Little finger*
**Rolandic operculum**	**L**	**−44**	**−8**	**18**	**83**	**15.06**	**5.31**
Postcentral gyrus	L	−58	−6	18		11.53	4.88
**Postcentral gyrus**	**L**	**−42**	**−20**	**60**	**164**	**13.33**	**5.12**
-	L	−32	−18	62		11.97	4.94

*Side indicates hemisphere (R = right, L = left), cluster size indicates N voxels, T indicates peak t-values, Z indicates peak z-values. Entries without brain region name labels indicate sub-peak within the cluster named above*.

**Table 3:** **Activated clusters from contrasting anlysis (finger stimulation vs. no stimulation) using univariate group GLM (*p* < 0.001 uncorrected, cluster size > 10)**.

		MNI coordinates					
Regions	Side	x	y	z	Voxels	T	Z
*Index finger*
**Postcentral gyrus**	**L**	**−48**	**−34**	**46**	**106**	**8.61**	**4.37**
Inferior parietal lobule	L	−58	−40	46		6.19	3.77
**Supramarginal gyrus**	**L**	**−58**	**−24**	**16**	**40**	**6.53**	**3.87**
*Middle finger*
**Postcentral gyrus**	**L**	**−40**	**−36**	**46**	**45**	**6.31**	**3.81**
-	L	−48	−32	48		6.29	3.80
-	L	−32	−36	48		4.63	3.23
*Ring finger*
	**No activation was found**						
*Little finger*
**Supramarginal gyrus**	**L**	**−64**	**−26**	**20**	**44**	**7.60**	**4.15**
-	L	−66	−28	28		7.07	4.02
**Supplementary motor area**	**L**	**2**	**6**	**54**	**42**	**6.57**	**3.88**

*Side indicates hemisphere (R = right, L = left), cluster size indicates N voxels, T indicates peak t-values, Z indicates peak z-values. Entries without brain region name labels indicate sub-peak within the cluster named above*.

## Discussion

In this study, we examined how vibrotactile information of stimulus locations was processed in the somatosensory system using both multi- and uni-variate analysis for the same dataset. Decoding analysis for stimulus location discrimination identified significant clusters in contralateral PPC and SMG, but not in S1, while contrasting finger stimulation vs. no stimulation activated distinct clusters mainly in the contralateral S1 (Figure 2). The discrepancy between these two analyses may reflect different roles for PPC and SMG (discrimination of the tactile stimulus) and S1 (detection of the tactile stimulus) in vibrotactile information processing. This observation favors the functional specialization for vibrotactile information in human somatosensory cortex, especially, hierarchical information processing of human somatosensory network assuming that tactile information is relayed along the serial pathway. Our results suggest that S1 is involved in more perceptual aspects of vibrotactile stimulus recognition and the adjacent brain regions (i.e., PPC and SMG) are involved in higher-level processing such as discrimination of stimulus locations.

**Figure 5:**
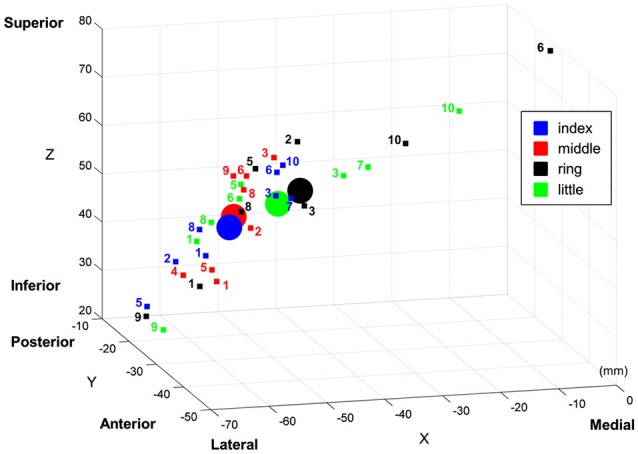
**“Center of mass” coordinates of the each finger representation in MNI space within primary somatosensory cortical area (S1)**. The “center of mass” coordinates of individual participant are marked with small squares, and the averaged “center of mass” coordinates are marked with circles. Note that the text next to individual “center of mass” indicates its participant number.

One of the key observations in the present study is that cortical regions activated by the discrimination of stimulus locations and the contrast of vibrotactile stimulation against no stimulation did not coincide: the stimulus location discrimination analysis identified PPC and SMG, and the contrast analysis identified S1. Based on the observation of this discrepancy, we claimed the hierarchical functional specialization for tactile information in the somatosensory cortex. A previous PET study has obtained similar results and suggested the hierarchical processing of tactile shape information (Bodegård et al., 2001). Their results showed that the somatosensory shape process initially took place in BA 3b and 1, and took an intermediate step through BA 2. SMG and intraparietal sulcus were implicated in more elaborate tactile processing as a final step. In an fMRI study, Reed et al. reported a functional hierarchy in somatosensory cortical areas such that sensorimotor areas were implicated in more perceptual aspects of tactile object recognition and inferior parietal regions including SMG were implicated in higher-level somatosensory processing (Reed et al., 2004). Then, how do we explain this discrepancy? In principle, there are two alternative explanations for these dissimilar activation patterns for location discrimination and stimulus presence. On one hand, this can be a statistical effect of our analysis such that the individual variations in S1 are too large for decoding finger locations with sufficient significance. On the other hand, the physiology in S1 does not provide sufficient variability across the participants and the discriminative signal is nevertheless too low to be significant; PPC and SMG are required for sufficient discrimination. To further investigate these two explanations, we examined individual variations in S1 responses. We computed the coordinates of the “center of mass” of each finger representation in the MNI coordinate (Figure 5), following the computation procedure described by Martuzzi et al. (2014). The “center of mass” coordinate of each stimulated finger was computed by means of a *t*-contrast over the HRF regressors of individual finger and they were mapped in the 3-dimensional MNI space. This individual GLM analysis showed the equal number (eight out of ten participants) of significant finger representations for each stimulated finger. In addition, we observed a pattern that the representation of index finger was located to a more lateral position while the little finger was located to a more medial position. This sequential pattern of the individual fingers within S1 is in agreement with previous finger somatotopy studies (Nelson and Chen, 2008; Schweizer et al., 2008; Martuzzi et al., 2014). It is noticeable that the individual “center of mass” coordinates of each finger were widely distributed over S1. Average center of mass of the middle and index finger are close together and the average center of mass of the ring and the little finger are reversed. This observation supports the assumption that the individual variations in S1 may be too large for decoding finger locations with sufficient significance, thus supporting the first explanation. Even though the individual GLM analysis revealed an equal number of significant BOLD-activation across the participants for each of the fingers, group GLM analyses did not identify significant activations in S1 for the ring and little finger. This result was probably caused by a larger spread of the individual BOLD-activation peak voxel for the little and the ring finger compared to the more clustered appearance of the individual peak voxel for the index and the middle finger. Moreover, the equal number of individual significant activations for each finger gives additional evidence that the missing significance of the group GLM for the ring and little finger is not based on a perceptual difference, nor on missing individual BOLD activation, but on the larger spatial distribution of the individual peak voxels for the ring and little fingers.

Classification patterns in the decoding analysis are also noteworthy. First, the misclassification rates were evenly distributed over the off-diagonal entries in the confusion matrix (Figure 4). Based on a well-established finding about organization of S1, one would expect to find a higher misclassification rate for adjacent fingers to the stimulated finger. We also had expected that the misclassification would tend towards the neighboring finger, but such a tendency was not observed. Our observation indicates that stimulus location information in PPC and SMG is not likely to be encoded in a sequential manner. Second, the classification performance combining all the voxels from the searchlight clusters was not significantly higher compared to the performance with each separate cluster. Since the more distinctive voxel response patterns were expected using all the voxels spanning both clusters, higher decoding accuracy was also expected. However, our results did not show significant difference between these cases. Further investigations are needed, but this observation in the present study demonstrates a possibility that encoded tactile information in PPC and SMG are characterized with different patterns.

Our results clearly showed the feasibility to decode passive vibrotactile stimulus locations across fingers using voxel response patterns and the contralateral PPC and SMG play a role in a tactile stimulus location decoding. Even though cortical areas that subserve tactile pattern discrimination have yet to be completely characterized, a number of studies have reported the involvement of both PPC and SMG areas in tactile sensation discrimination (Francis et al., 2000; Bodegård et al., 2001; Li Hegner et al., 2007, 2010). Human neuroimaging studies reported activations in PPC and SMG during the tactile pattern discrimination task in a magnetoencephalography (MEG) study (Li Hegner et al., 2007) and in an fMRI study (Van Boven et al., 2005; Li Hegner et al., 2010). Van Boven et al. showed that the anterior part of the SMG in the inferior parietal cortex is involved in tactile form and location processing (Van Boven et al., 2005). In a similar vein, it was reported that PPC made a contribution in remapping tactile information receiving from primary somatosensory cortical activities (Azañón et al., 2010; Bufalari et al., 2014). An electroencephalogram (EEG) study of neural correlates of somatosensory illusions showed that PPC activities were correlated with the tendency to solve conflict between tactile and proprioceptive inputs while S1 activities were solely related to illusory perception (Bufalari et al., 2014).

Despite convergence of the present finding with other tactile imaging studies, our findings can be limited by the effect of the spatial smoothing with a 4 mm (FWHM) Gaussian kernel because the higher spatial resolution of the data may be required for a successful GLM decoding analysis (Mikl et al., 2008). To eliminate this potential risk due to the spatial smoothing, we performed the “single finger against all other fingers” group GLM analysis and the “stimulated finger decoding” searchlight analysis again with a 2 mm of FWHM that was used in the previous somatotopy study (Martuzzi et al., 2014). In spite of the variation of the Gaussian kernel size, however, we obtained the same results. Group GLM analysis did not find any significant brain region and searchlight analysis identified PPC and SMG. Specifically, searchlight analysis with 2 mm smoothing showed that the peak coordinates were similar but the significant cluster size and the peak *t*-value were reduced. Hence, the stability of the statistical results in the GLM as well as in the MVPA under 4 mm as well as under 2 mm smoothing suggests an equivalent resolvability of the finger representations even under the larger smoothing kernel.

## Conclusion

The present study explored the brain activity in response to 200 Hz of vibrotactile stimuli applied to the fingers to investigate how vibrotactile sensory information is represented in the hierarchical somatosensory system. In particular, we examined the fMRI signals using both the multivariate searchlight analysis and the univariate GLM analysis. We statistically assessed each set of multi-voxel patterns in terms of discrimination ability for finger locations. The feasibility of decoding finger location information was verified with significant higher accuracy than a chance level. Our decoding analysis revealed that PPC and SMG (not in S1) contained significant multi-voxel sets for discriminating finger locations. Contrasting vibrotactile stimulus vs. no stimulus, significant activations were observed in S1 (not in PPC and SMG). In spite of the inadequacy of group GLM analysis, therefore, results underpin the hierarchical view of the organization of the somatosensory system.

Our findings generally support that S1 is essential for reflecting touch sensation. For the purpose of a successful discrimination between fingers, however, the subsequent, “next level” brain regions, namely PPC and SMG need to be recruited. Future studies will examine whether requiring such “higher” processing regions for decoding also hold outside the somatosensory system.

## Conflict of interest statement

The authors declare that the research was conducted in the absence of any commercial or financial relationships that could be construed as a potential conflict of interest.
